# Plasma soluble erythropoietin receptor is decreased during sleep in Andean highlanders with Chronic Mountain Sickness

**DOI:** 10.1152/japplphysiol.00107.2016

**Published:** 2016-04-28

**Authors:** Francisco C. Villafuerte, Noemí Corante, Cecilia Anza-Ramírez, Rómulo Figueroa-Mujíca, Gustavo Vizcardo-Galindo, Andy Mercado, José Luis Macarlupú, Fabiola León-Velarde

**Affiliations:** ^1^Laboratorio de Fisiología Comparada, Departamento de Ciencias Biológicas y Fisiológicas, Facultad de Ciencias y Filosofía, Universidad Peruana Cayetano Heredia, Lima, Peru

**Keywords:** soluble erythropoietin receptor, Chronic Mountain Sickness, excessive erythrocytosis, sleep, Andes

## Abstract

Excessive erythrocytosis (EE) is the main sign of Chronic Mountain Sickness (CMS), a highly prevalent syndrome in Andean highlanders. Low pulse O_2_ saturation (SpO_2_) during sleep and serum androgens have been suggested to contribute to EE in CMS patients. However, whether these factors have a significant impact on the erythropoietin (Epo) system leading to EE is still unclear. We have recently shown that morning soluble Epo receptor (sEpoR), an endogenous Epo antagonist, is decreased in CMS patients suggesting increased Epo availability (increased Epo/sEpoR). The present study aimed to characterize the nocturnal concentration profile of sEpoR and Epo and their relationship with SpO_2_, Hct, and serum testosterone in healthy highlanders (HH) and CMS patients. Epo and sEpoR concentrations were evaluated every 4 h (6 PM to 6 AM) and nighttime SpO_2_ was continuously monitored (10 PM to 6 AM) in 39 male participants (CMS, *n* = 23; HH, *n* = 16) aged 21-65 yr from Cerro de Pasco, Peru (4,340 m). CMS patients showed higher serum Epo concentrations throughout the night and lower sEpoR from 10 PM to 6 AM. Consequently, Epo/sEpoR was significantly higher in the CMS group at every time point. Mean sleep-time SpO_2_ was lower in CMS patients compared with HH, while the percentage of sleep time spent with SpO_2_ < 80% was higher. Multiple-regression analysis showed mean sleep-time SpO_2_ and Epo/sEpoR as significant predictors of hematocrit corrected for potential confounders (age, body mass index, and testosterone). Testosterone levels were associated neither with Hct nor with erythropoietic factors. In conclusion, our results show sustained erythropoietic stimulus driven by the Epo system in CMS patients, further enhanced by a continuous exposure to accentuated nocturnal hypoxemia.

## Abstract (precis)

*Andean highlanders suffering from Chronic Mountain Sickness (CMS) show consistently lower levels of plasma soluble erythropoietin (Epo) receptor (sEpoR) and higher Epo-to-EpoR ratios (Epo/sEpoR) during sleep compared with their healthy counterparts. This indicates higher blood Epo availability in CMS patients and continuous nocturnal erythropoietic stimulus. Additionally, morning Epo/sEpoR and mean sleep-time SpO_2_ are independent main predictors of Hct. These findings support the role of the Epo system in the development of excessive erythrocytosis in CMS*.

## NEW & NOTEWORTHY

*Andean highlanders suffering from Chronic Mountain Sickness (CMS) show consistently lower levels of plasma soluble erythropoietin (Epo) receptor (sEpoR) and higher Epo-to-EpoR ratios (Epo/sEpoR) during sleep compared with their healthy counterparts. This indicates higher blood Epo availability in CMS patients and continuous nocturnal erythropoietic stimulus. Additionally, morning Epo/sEpoR and mean sleep-time SpO*_*2*_ *are independent main predictors of Hct. These findings support the role of the Epo system in the development of excessive erythrocytosis in CMS*.

chronic mountain sickness (CMS) is a highly prevalent syndrome among Andean highlanders. The prevalence of this condition increases with age and altitude (16, 27, 35, 40) and affects 16–20% of the population living permanently above 4,000 m, particularly men and postmenopausal women (19, 25, 27). CMS is characterized by excessive erythrocytosis (EE, Hb concentration ≥21.3 g/dl in men and ≥18.6 g/dl in women), and it is frequently accompanied by exaggerated hypoxemia (17, 20, 25, 26). The associated symptoms are headaches, dizziness, breathlessness, tinnitus, palpitations, sleep disturbances, mental and physical fatigue, and confusion (17, 20). Pulmonary hypertension is also commonly associated with the condition (21, 28, 30, 37), and people affected by CMS often suffer from vascular dysfunction and heart failure in early adulthood (17, 24, 33, 38), possibly due to increased blood viscosity (15).

Prolonged hypoxia exposure represents the underlying stimulus for EE and CMS (20, 23, 44); however, the fundamental pathophysiological mechanism is still debatable. It is not clear why only some individuals chronically exposed to high altitude (HA) develop this syndrome.

Sleep-disordered breathing leading to accentuated hypoxemia has been proposed as a possible factor triggering EE. However, studies of sleep abnormalities at HA show discordant results. Spicuzza et al. (36) showed no differences in the number and duration of apneas or hypopneas between patients with EE and healthy controls, while Julian et al. (10) have reported that young male residents with EE show a higher frequency of apneas (central and obstructive) and hypopneas during REM sleep and a greater apnea-hypopnea index. Despite controversy on the occurrence of sleep abnormalities, both studies agree on the presence of lower nocturnal pulse O_2_ saturation (SpO_2_) in subjects with EE compared with their healthy counterparts, which is consistent with other studies of nocturnal SpO_2_ in HA polycythemic patients (8, 14, 31).

Also, whether accentuated hypoxemia during sleep has a significant impact on circulating erythropoietin (Epo) concentration that could explain the occurrence of EE in CMS patients is still controversial. Despite greater nighttime hypoxemia, most studies have shown similar morning Epo concentration in patients with EE compared with healthy highlanders (HH) (6, 10, 18, 39). In contrast, Bernardi et al. (1) reported in a small number of highlanders of Cerro de Pasco (4,340 m) that the hormone is slightly but consistently higher in CMS patients during day and night compared with healthy controls. However, high variability in serum Epo between subjects with EE suggests other mechanisms involved in the development of an excessive erythropoietic response. The soluble Epo receptor (sEpoR), an endogenous Epo antagonist (22, 29), has been suggested to play a role as a physiological regulator of erythropoiesis (11, 39). We have recently shown that EE is strongly associated with low circulating morning sEpoR values and therefore with high Epo-to-sEpoR ratios (Epo/sEpoR), leading to elevated plasma Epo availability and thereby a stronger stimulus for erythropoiesis (39). However, it is still unknown if differences in sEpoR concentration during sleep and its relationship with Epo and SpO_2_ are associated with EE.

In addition, other hormonal factors, such as testosterone, have been proposed as possible contributors to the development of EE and CMS (6). Elevated morning serum testosterone has been reported to be associated with EE in Andean highlanders (6, 7). However, whether serum testosterone contributes to the Epo system in the excessive production of erythrocytes in CMS is yet to be evaluated.

The aim of the present study was to characterize the concentration profile of sEpoR and serum Epo during nighttime and their relationship with SpO_2_ and hematocrit (Hct) in CMS patients and HH. Additionally, we aimed to assess the association between testosterone concentration and Epo and sEpoR in early morning.

## METHODS

The study was approved by the Institutional Ethics Committee (CIE) of Universidad Peruana Cayetano Heredia (Lima, Peru). Participants provided written, informed consent.

### 

#### Study population.

Thirty-nine male participants (CMS, *n* = 23; HH, *n* = 16) in the age range from 21 to 65 yr from Cerro de Pasco, Peru, at 4,340 m, were studied. Exclusion criteria were the presence of pulmonary, cardiovascular, or renal disease, recent phlebotomy, journeys to lower altitude for more than 7 days during the previous 6 months, and employment in mining activities.

A sample size of 36 was calculated on the basis of Epo measurements from a previous study in the same population (32). This erythropoietic marker was employed since it is the study parameter known to show the largest variability. Taking into account a potential 20% loss of participants due mainly to missing data or extreme values, we evaluated 45 individuals. Finally, 39 highlanders with complete data were included in the analysis.

#### Study procedures.

Participants underwent a clinical examination and answered a general health and a CMS score questionnaire (17). CMS was confirmed in participants with Hct > 63% (Hb > 21 g/dl) and a CMS score ≥6 according to international consensus (17). Hct was determined by microcentrifugation using a small blood sample obtained from a puncture on the fingertip. Nighttime SpO_2_ was continuously monitored from 10 PM to 6 AM through a wrist pulse oximeter (WristOx_2_ Model 3150; Nonin, Plymouth, MN). Blood samples for Epo and sEpoR determination were taken every 4 h from 6 PM to 6 AM. Specific sandwich enzyme immunoassay kits were used for serum Epo (DRG International, Springfield, NJ) and sEpoR (USCN Life Science, Houston, TX) concentration quantification. The standard sample storage and analysis procedure described by the manufacturer was followed for each kit running each sample in duplicate. Serum from the 6 AM sample was also used for the assessment of serum iron, ferritin, transferrin, and free and total testosterone, which were analyzed at Medlab clinical laboratories (ISO 9001:2000), Lima, Peru.

#### Statistical analysis.

Differences between groups in the general characteristics of the study participants were obtained through unpaired *t*-tests, when data met requirements of normality and homocedasticity, or Wilcoxon rank sum tests if otherwise. Generalized estimating equations were used to evaluate the effect of time, group (CMS or HH), and their interaction on Epo, sEpoR, the Epo/sEpoR ratio, and SpO_2_ profiles, adjusted for potential confounders (age, BMI, and testosterone concentration). In addition, differences between time points within each group and between groups at each time point were estimated by the use of postregression analysis. Finally, correlation analysis was performed to evaluate bivariate associations, and multiple linear regressions were used to assess the association between Hct and SpO_2_ and markers of the Epo system (Epo, sEpoR, and Epo/sEpoR ratio) in the presence of potential confounders. Only multiple regressions that met requirements of linearity, normality, and homocedasticity were included, and variables with no linearity were categorized. A significance level of 0.05 was used.

#### Study variables.

CMS score was determined in agreement with the 2005 Consensus Statement on Chronic and Subacute HA Diseases (17). Awake SpO_2_ corresponds to the value obtained from clinical examination that took place around 6 PM while the person was at rest and is calculated as the mean of two measurements taken 2 min apart. Basal presleep SpO_2_ measurements at 10 PM were retrieved from the continuous nighttime monitoring, readings from 5 min after the device had been placed and the patient was in bed were used, and a mean value was calculated using two readings 2 min apart. Mean values for sleep-time SpO_2_ from 2 AM and 6 AM used for the bivariate correlations were calculated in the same manner. Mean sleep-time SpO_2_ was calculated using measurements collected every 30 s throughout the time the patient was asleep. Continuous nighttime readings were also used to calculate mean SpO_2_ for every hour of sleep and for the sampling period. The percentage of sleep time spent with SpO_2_ below 80% was calculated using hours of sleep spent below this value in relation to total sleep time (TST). The cutoff point was chosen in accordance with previous literature where SpO_2_ below 80% has been described as a threshold for the stimulation of Epo production (2, 36). For multiple-regression analysis, BMI was dichotomized into normal and overweight using a cutoff value of 25 kg/m^2^ in agreement with World Health Organization guidelines (45), and age was categorized using 45 yr as a general cutoff value for the onset of increased morbidity (4, 25, 27).

## RESULTS

### 

#### General characteristics.

Mean age and BMI were similar between study groups. As previously reported (20), CMS score and Hct were significantly higher in CMS patients compared with HH, while awake (6 PM) SpO_2_ was significantly lower. No differences were found in serum iron, ferritin, or transferrin concentration or transferrin saturation between groups (Table 1).

**Table 1.** General characteristics of study participants

	Healthy Highlanders	CMS Patients
Age, yr	40.1 ± 2.83	44.26 ± 2.72
BMI, kg/m^2^	25.4 ± 0.70	26.07 ± 0.73
SpO_2_, %	86.9 ± 1.02	82.33 ± 1.17*
Hct, %	52.7 ± 0.70	69.82 ± 1.13†
CMS score	2.4 ± 0.60	8.52 ± 0.78†
Serum iron, μg/dl	115.6 ± 16.54	106.03 ± 10.01
Serum ferritin, ng/ml	102.8 ± 20.61	96.13 ± 20.76
Transferrin, mg/dl	257.7 ± 7.01	267.00 ± 12.28
Transferrin saturation, %	45.9 ± 5.92	42.52 ± 4.74

Values are expressed as means ± SE; healthy highlanders, *n* = 16; Chronic Mountain Sickness (CMS) patients, *n* = 23. BMI, body mass index; SpO_2_, pulse O_2_ saturation.

* *P* < 0.05,

† *P* < 0.001.

#### SpO_2_.

**Fig. 1.**
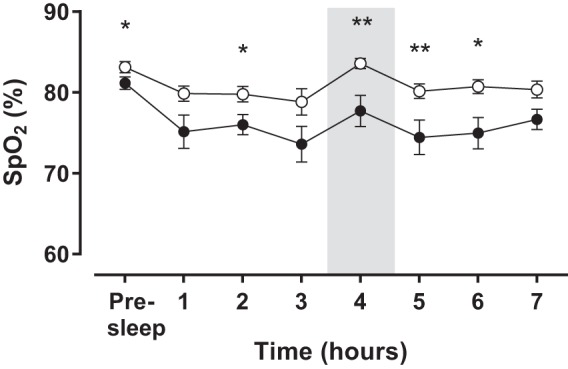
Time course of nocturnal pulse oxygen saturation (SpO_2_) in patients with Chronic Mountain Sickness (●, *n* = 23) and healthy highlanders (○, *n* = 16). The figure shows SpO_2_ while the patients were awake in bed and for each hour of sleep. The period highlighted in grey corresponds to the sampling time for which patients were awakened. Values are expressed as means ± SE. *P < 0.05, **P < 0.01.

**Fig. 2.**
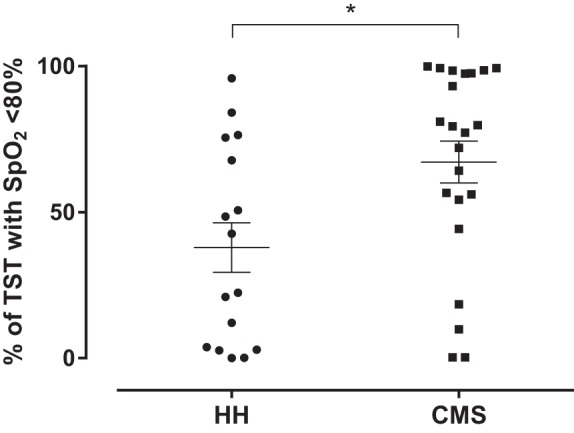
Percentage of total sleep time (TST) spent with pulse oxygen saturation (SpO_2_) below 80% in patients with Chronic Mountain Sickness (CMS, *n* = 23) and healthy highlanders (HH, *n* = 16). The figure shows a scatter of individual values for each group. Horizontal lines represent means ± SE. *P < 0.05.

Basal presleep (10 PM) SpO_2_ and SpO_2_ throughout the night were consistently lower in CMS patients compared with HH, including the sampling period (Fig. 1). Moreover, CMS patients showed significantly lower mean sleep-time SpO_2_ (*P* < 0.01). The percentage of TST spent with SpO_2_ below 80% was significantly greater in CMS patients compared with HH (Fig. 2). In additional correlation analysis, awake SpO_2_ (6 PM) as well as sleep-time measurements from 2 and 6 AM and mean sleep-time SpO_2_ showed significant negative associations with Hct (ρ = −0.59, *P* < 0.01; ρ = −0.44, *P* < 0.01; ρ = −0.42, *P* < 0.01; and ρ =−0.56, *P* < 0.001, respectively). Also, awake (6 PM) and 2 AM SpO_2_ showed significant negative association with the CMS score (ρ = −0.55, *P* < 0.001; ρ = −0.47, *P* < 0.01, respectively). Mean sleep-time SpO_2_ showed a tendency for a correlation with 6 AM (awake) Epo (ρ = −0.31, *P* = 0.05).

#### Epo, sEpoR, and Epo/sEpoR ratio.

**Fig. 3.**
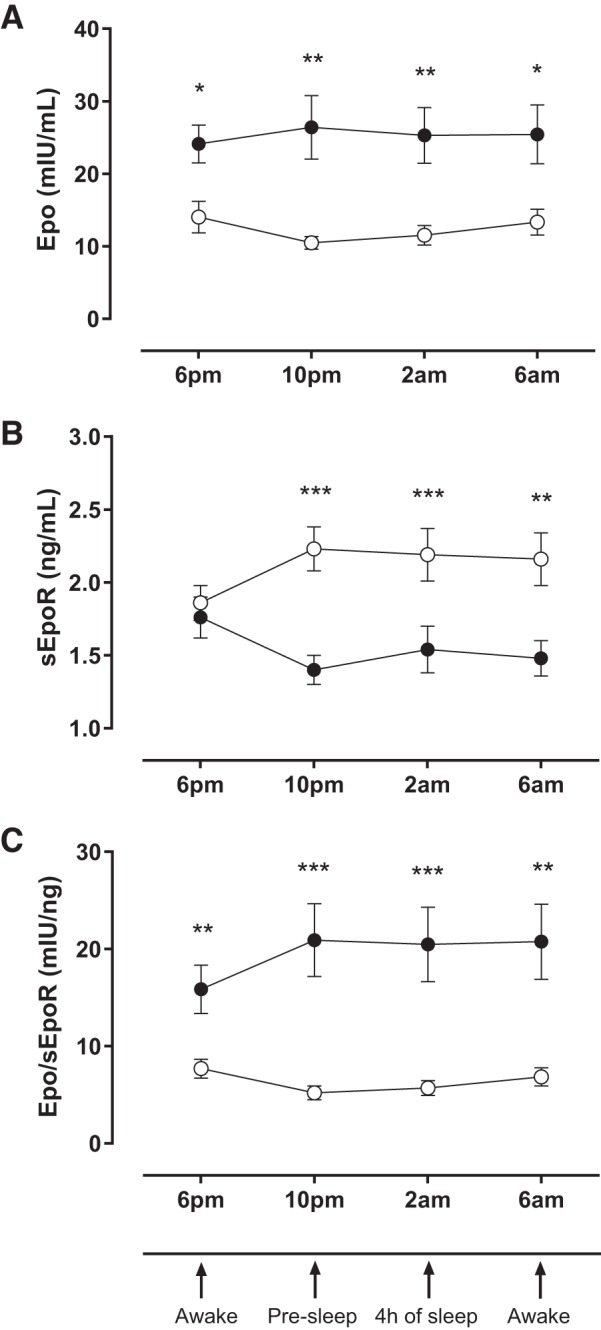
Time course of erythropoietin (Epo), serum soluble Epo receptor (sEpoR), and the Epo-to-sEpoR (Epo/sEpoR) ratio in patients with Chronic Mountain Sickness (●, *n* = 23) and healthy highlanders (○, *n* = 16). The figure shows Epo (*A*) and sEpoR (*B*) concentrations and the Epo/sEpoR ratio (*C*) of each group at every time point. Arrows on parallel *x*-axis each point at specific time points on Epo, sEpoR, and Epo/sEpoR measurements and corresponding time points at 10 PM (presleep) and 2 AM (4 h of sleep) from Fig. 1. Values are expressed as means ± SE. *P < 0.05, **P < 0.01, ***P < 0.001.

Epo concentration, sEpoR, and the Epo/sEpoR ratio were significantly different between groups throughout the measurements, except for the receptor at 6 PM (Fig. 3). Comparisons between time points within each group showed a marked increase in the receptor from 6 to 10 PM in the HH group (*P* = 0.011), while values decreased significantly between these time points in the CMS group (*P* = 0.046). Accordingly, a significant interaction was found between time and group effects for the receptor (Fig. 3*B*). Also, in the HH group, the Epo/sEpoR ratio showed significantly higher values at 10 PM and 2 AM compared with 6 PM (*P* = 0.002 and *P* = 0.041, respectively) (Fig. 3*C*). Finally, no differences were found in Epo between time points within each group (Fig. 3*A*).

#### Testosterone.

**Fig. 4.**
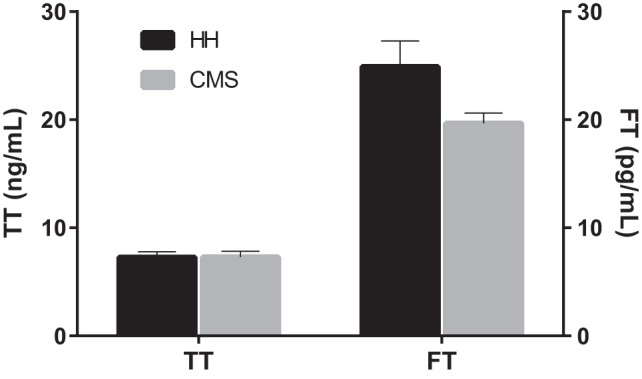
Serum total testosterone (TT) and free testosterone (FT) concentration in patients with Chronic Mountain Sickness (CMS, *n* = 23) and healthy highlanders (HH, *n* = 16). Values are expressed as means ± SE.

Morning total testosterone and free testosterone concentrations were not significantly different between the CMS and HH groups either in the study subjects (Fig. 4) or in a larger sample evaluated for this parameter (*n*: HH = 47 and CMS = 31). Also, no significant correlations were observed between total or free testosterone and morning Epo, sEpoR, or the Epo/sEpoR ratio.

#### Regression.

Multiple-regression analysis showed mean sleep-time SpO_2_ and the Epo/sEpoR ratio as significant predictors of Hct corrected for potential confounders (Table 2). Similar results were obtained when regressions included Epo or the sEpoR as predictors (data not shown).

**Table 2.** Multiple-linear regression models for prediction of hematocrit

Independent Variables	β	SE	*P*	Model *R*^2^
*Model A*				
Mean sleep-time SpO_2_	−0.51	0.20	0.017	0.47
Epo/sEpoR	0.31	0.08	0.001	
Age >45 yr	2.70	2.67	0.320	
BMI >25 kg/m^2^	3.82	2.59	0.151	
Free testosterone	−0.02	0.14	0.871	
*Model B*				
Mean sleep-time SpO_2_	−0.50	0.20	0.022	0.47
Epo/sEpoR	0.32	0.07	0.000	
Age >45 yr	2.93	2.74	0.294	
BMI >25 kg/m^2^	4.23	2.62	0.117	
Total testosterone	0.21	0.57	0.708	

*Model A*, multiple linear regression for prediction of hematocrit including mean sleep-time SpO_2_, Epo/sEpoR ratio, being over 45 yr of age, having over 25 kg/m^2^ BMI, and free testosterone as independent variables; *model B*: multiple linear regression for prediction of hematocrit including mean sleep-time SpO_2_, Epo/sEpoR ratio, being over 45 yr of age, having over 25 kg/m^2^ BMI, and total testosterone as independent variables. SpO_2_, pulse O_2_ saturation; BMI, body mass index. β, regression coefficient; SE, standard error; *R*^2^: coefficient of determination.

## DISCUSSION

This is the first study to report the temporal profile of plasma sEpoR during sleep in CMS patients and HH and its relationship with circulating Epo and SpO_2_.

The results from nighttime measurements of Epo, the receptor, and the Epo/sEpoR ratio from the present study complement our previous findings on the association of low morning sEpoR with EE (39). Here we show that besides lower morning plasma sEpoR, nighttime concentrations are also significantly lower in CMS patients compared with HH. Additionally, differentiated time-dependent variations in sEpoR were observed in the HH group and CMS patient group. Results show that while in HH the receptor increases from 6 to 10 PM and remains elevated through the night, it decreases in CMS patients and remains low until morning. The behavior of the receptor suggests the presence of rhythmicity in its secretion, being different between groups, although measurements throughout the entire day are needed to evaluate the possibility of a circadian rhythm in HH and possible alterations in CMS patients. Similar differences between groups were observed for the Epo/sEpoR ratio. In congruence with our previous work (39), in the present study the Epo/sEpoR ratio showed higher values in CMS patients in the morning and throughout the night compared with HH, indicating a constant increased availability of Epo from 6 PM to 6 AM and therefore a continuous erythropoietic stimulus in these patients.

Different studies have shown that SpO_2_ during daytime is lower in patients with EE compared with HH and that this difference is maintained throughout the night (8, 13, 36). In agreement, we found lower daytime and nighttime SpO_2_ in CMS patients compared with HH, and a similar drop in SpO_2_ could be observed in both groups during sleep. However, despite similar absolute nocturnal desaturation in HH and CMS patients, the latter begin the night with lower values compared with their healthy counterparts, so that the drop in SpO_2_ during sleep is enough to reach values below 80%, a threshold known to trigger the erythropoietic response (2). Spicuzza et al. (36) showed that patients with CMS spent 38% of the night with SpO_2_ values between 76 and 80%, while healthy controls spent most of the night above 81%. Accordingly, we found that the percentage of TST spent with SpO_2_ values below 80% in CMS patients is nearly twice the values observed in HH, and also a strong negative association between mean sleep-time SpO_2_ and Hct was observed. Thus it would be expected that a greater exposure time to accentuated hypoxemia further stimulates the Epo system and contributes to the development of EE. However, most studies have reported similar morning serum Epo concentration in HH and patients with EE (6, 18, 39). Nevertheless, since these studies used measurements from a single time point, the occurrence of variations in the hormone levels throughout 24 h was not considered. In this sense, studies with repeated measurements provide more precise comparisons than cross-sectional studies, yielding more reliable results. Additionally, longitudinal studies shed light into potential differences between groups at several times of day and into diurnal or nocturnal variations in the hormone, and in the Epo system as whole, that might contribute to the development of EE.

Although there is some disagreement between reports of variations of Epo during 24 h (9, 12, 34), some studies reported a circadian rhythm for the hormone, both in sea level inhabitants (3, 5, 12, 42) and HA healthy natives (1), consistently showing a nadir in the morning and a zenith in the afternoon. To evaluate diurnal and nocturnal variations of Epo, Bernardi et al. (1) studied a small number of HA dwellers from Cerro de Pasco with and without EE by taking measurements every 4 h beginning at 8 AM. The study showed that circadian rhythm was disrupted in subjects with EE, with no variations between daytime and nighttime Epo concentration, as opposed to the 40–60% variation observed in HH. At every time point, Epo was higher in patients with EE compared with HH, and there was neither morning nadir nor distinguishable zenith, although great variability between subjects was observed. In agreement with Bernardi et al., we found higher Epo concentration throughout the measurements in CMS patients compared with HH, although the highest variability in both groups could be observed in the morning. Also, we found no signs of rhythmicity in Epo secretion either in CMS patients or in HH, probably because of lack of daytime measurements since the peak and the nadir of Epo concentration have been reported around 4–8 PM and 8–12 AM, respectively (12, 42), outside our sampling period.

Some potential limitations can be identified in our study. First, subjects had to be awakened during the night for sample-drawing procedure. To overcome this shortcoming, we reduced the sampling period to a minimum, as well as disturbances caused by sound or light. Additionally, this period was not included in the calculation of nighttime hypoxemia-related variables. Second, it was not possible to evaluate a circadian rhythm of Epo and the receptor, since no complete daytime measurements were obtained. However, our results of nighttime values of the Epo system variables shed light into the behavior of sEpoR and set a precedent for the study of circadian rhythmicity of the Epo system as a whole.

The findings of the present investigation suggest that higher Epo concentration during the night might play a role in maintaining the erythropoietic stimulus responsible for EE. However, high between-subject variability in Epo concentration, especially in the CMS group, suggests that there are other mechanisms promoting the excessive erythropoietic response. Our findings of higher Epo/sEpoR ratio in CMS patients compared with HH at every time point, in contrast with controversial results on daytime Epo, indicate that the erythropoietic drive in CMS might be better explained in terms of Epo availability (Epo/sEpoR ratio) rather than Epo concentration alone. Additionally, we show that morning Epo/sEpoR ratio and mean sleep-time SpO_2_ are independent predictors of Hct and that the ratio is a better predictor compared with serum Epo.

Our results also indicate that neither is testosterone a significant predictor of Hct nor was a relationship observed between the hormone and the presence of CMS, in contrast with results from previous studies by Gonzales and colleagues (6, 7). Also, no significant association was found between total or free serum testosterone concentration and Epo, sEpoR, or the Epo/sEpoR either in the bivariate analysis or the multiple regressions including mean sleep-time SpO_2_, age, and BMI.

In conclusion, the present study shows that sEpoR is consistently lower in CMS patients during nighttime, leading to an increased Epo/sEpoR ratio, which suggests a continuous elevated Epo availability and consequently a stronger stimulus for erythropoiesis, without a significant contribution from serum androgens. These, together with our previous findings, suggest that CMS patients show a sustained erythropoietic stimulus driven by the Epo system, further enhanced by the continuous exposure to accentuated hypoxemia during sleep.

## GRANTS

This research was supported by a Wellcome Trust Public Health and Tropical Medicine Fellowship (097275/Z/11/Z) to F. C. Villafuerte.

## DISCLOSURES

No conflicts of interest, financial or otherwise, are declared by the author(s).

## AUTHOR CONTRIBUTIONS

F.C.V. and F.L.V. conception and design of research; F.C.V., N.C., C.A.-R., R.F.-M., G.V.-G., A.M., and J.L.M. performed experiments; F.C.V., N.C., C.A.-R., and R.F.-M. analyzed data; F.C.V., N.C., C.A.-R., R.F.-M., G.V.-G., A.M., J.L.M., and F.L.V. interpreted results of experiments; F.C.V. and N.C. prepared figures; F.C.V. and N.C. drafted manuscript; F.C.V., N.C., C.A.-R., R.F.-M., G.V.-G., A.M., J.L.M., and F.L.V. edited and revised manuscript; F.C.V., N.C., C.A.-R., R.F.-M., G.V.-G., A.M., J.L.M., and F.L.V. approved final version of manuscript.
